# Draft Genome Sequence of *Bacillus cereus* LCR12, a Plant Growth–Promoting Rhizobacterium Isolated from a Heavy Metal–Contaminated Environment

**DOI:** 10.1128/genomeA.01041-16

**Published:** 2016-09-29

**Authors:** Eleonora Egidi, Jennifer L. Wood, Elizabeth Mathews, Edward Fox, Wuxing Liu, Ashley E. Franks

**Affiliations:** a Department of Physiology, Anatomy and Microbiology, La Trobe University, Bundoora, Victoria, Australia; b CSIRO Animal, Food and Health Sciences, Werribee, Victoria, Australia; c Key Laboratory of Soil Environment and Pollution Remediation, Institute of Soil Science, Chinese Academy of Sciences, Nanjing, China

## Abstract

*Bacillus cereus* LCR12 is a plant growth–promoting rhizobacterium, isolated from a heavy metal–contaminated environment. The 6.01-Mb annotated genome sequence provides the genetic basis for revealing its potential application to remediate contaminated soils in association with plants.

## GENOME ANNOUNCEMENT

Given their ability to increase the availability of mineral nutrients, produce plant growth–stimulating compounds, and protect against soil-borne pathogenic fungi (1), plant growth–promoting rhizobacteria (PGPR) are widely used as bioinoculants to support survival and development of plants under various stressing conditions, such as heavy metal contamination of soil (2).

Species belonging to the genus *Bacillus* are predicted to play an important role in the successful survival and growth of plants in polluted soil by alleviating the xenobiotic toxicity and supplying nutrients (3). In order to provide further insight into the physiology, metabolic potential, and possible future applications of plant growth–promoting rhizobacteria, we report the draft genome of *Bacillus cereus* LCR12, a PGPR isolated from the rhizosphere of the heavy metal hyperaccumulator *Carpobrotus rossii* (Haw.) Schwantes grown in a Cd-contaminated environment (4). The strain was found to have an MIC of 50 mg·L^−1^ for Cd on agar plates.

The strain was grown in cell culture, and the total genomic DNA was extracted from purified *B. cereus* LCR12 and converted to sequencing libraries using the Nextera XT DNA library preparation kit (Illumina). Libraries were normalized and pooled before sequencing on an Illumina MiSeq with 2 × 300 paired-end reads. For each isolate, the A5-miseq pipeline (5) was used to perform read trimming and correction, contig assembly, crude scaffolding, misassembly correction, and final scaffolding. No putative misassemblies were detected with this method. The scaffolds were then reordered with Mauve (6) using *B. cereus* ATCC 14579 as the reference genome (GenBank accession number NC_004722.1). Genome sequencing resulted in high coverage assemblies of the ~6-Mb genome (78-fold median coverage), which should represent most of the functional annotated genes and allow for comparative studies. The final number of contigs was 40 with an *N*_50_ value of 3,706,762.

The draft genome sequence of *B. cereus* LCR12 comprises 6,031,570 bp, with a G+C content of 34.8%. A total of 6,005 coding sequences were annotated with the RAST annotation system (7). Several predicted genes carried functions involved in the bacterium-plant interaction, such as genes encoding for siderophores (bacillibactin and petrobactin), which increase the availability and uptake of iron in plants, and genes involved in the exopolysaccharide and lipopolysaccharide biosynthesis (e.g., exopolysaccharide operon *EpsC-D*) for the formation of biofilms, a necessary prerequisite for the efficient colonization of the root surface (8). The LCR12 genome contains 109 genes involved in phosphorus metabolism, which may be responsible for making organic phosphate available to the plants (9). In addition, we found eight genes associated to the chitin metabolism, suggesting a possible role of LCR12 in the protection against fungal phytopathogens. Finally, we were able to identify several genes related to heavy metal resistance, including chromium, cadmium, arsenic, and copper, as well as antibiotics (e.g., multidrug resistance operon *mdtR*, efflux pumps *Lde*, *VanS*, and *VanR*), suggesting the ability of this strain to resist numerous environmental stresses.

### Accession number(s).

This whole-genome shotgun project has been deposited at DDBJ/ENA/GenBank under the accession number MCAX00000000. The version described in this paper is the first version, MCAX01000000.

## References

[1] Doornbos RF, van Loon LC, Bakker PA 2012 Impact of root exudates and plant defense signaling on bacterial communities in the rhizosphere. A review. Agron Sustain Dev 32:227–243. doi:10.1007/s13593-011-0028-y.

[2] Zhuang X, Chen J, Shim H, Bai Z 2007 New advances in plant growth-promoting rhizobacteria for bioremediation. Environ Int 33:406–413. doi:10.1016/j.envint.2006.12.005. 17275086

[3] Yu X, Li Y, Zhang C, Liu H, Liu J, Zheng W, Kang X, Leng X, Zhao K, Gu Y, Zhang X, Xiang Q, Chen Q 2014 Culturable heavy metal-resistant and plant growth promoting bacteria in V−Ti magnetite mine tailing soil from Panzhihua, China. PLoS One 9:e106618. doi:10.1371/journal.pone.0106618. 25188470 PMC4154735

[4] Zhang C, Sale PW, Doronila AI, Clark GJ, Livesay C, Tang C 2014 Australian native plant species *Carpobrotus rossii* (Haw.) Schwantes shows the potential of cadmium phytoremediation. Environ Sci Pollut Res Int 21:9843–9851. doi:10.1007/s11356-014-2919-3. 24777324

[5] Coil D, Jospin G, Darling AE 2015 A5-miseq: an updated pipeline to assemble microbial genomes from Illumina MiSeq data. Bioinformatics 31:587–589. doi:10.1093/bioinformatics/btu661. 25338718

[6] Darling AE, Tritt A, Eisen JA, Facciotti MT 2011 Mauve assembly metrics. Bioinformatics 27:2756–2757. doi:10.1093/bioinformatics/btr451. 21810901 PMC3179657

[7] Aziz RK, Bartels D, Best AA, DeJongh M, Disz T, Edwards RA, Formsma K, Gerdes S, Glass EM, Kubal M, Meyer F, Olsen GJ, Olson R, Osterman AL, Overbeek RA, McNeil LK, Paarmann D, Paczian T, Parrello B, Pusch GD 2008 The RAST server: Rapid Annotations using Subsystems Technology. BMC Genomics 9:75. doi:10.1186/1471-2164-9-75. 18261238 PMC2265698

[8] Ramey BE, Koutsoudis M, von Bodman SB, Fuqua C 2004 Biofilm formation in plant–microbe associations. Curr Opin Microbiol 7:602–609. doi:10.1016/j.mib.2004.10.014. 15556032

[9] Vessey JK 2003 Plant growth promoting rhizobacteria as biofertilizers. Plant Soil 255:571–586. doi:10.1023/A:1026037216893.

